# Bottom-Up Engineering of Biological Systems through Standard Bricks: A Modularity Study on Basic Parts and Devices

**DOI:** 10.1371/journal.pone.0039407

**Published:** 2012-07-20

**Authors:** Lorenzo Pasotti, Nicolò Politi, Susanna Zucca, Maria Gabriella Cusella De Angelis, Paolo Magni

**Affiliations:** 1 Dipartimento di Ingegneria Industriale e dell'Informazione, Università degli Studi di Pavia, Pavia, Italy; 2 Centro di Ingegneria Tissutale, Università degli Studi di Pavia, Pavia, Italy; Center for Genomic Regulation, Spain

## Abstract

**Background:**

Modularity is a crucial issue in the engineering world, as it enables engineers to achieve predictable outcomes when different components are interconnected. Synthetic Biology aims to apply key concepts of engineering to design and construct new biological systems that exhibit a predictable behaviour. Even if physical and measurement standards have been recently proposed to facilitate the assembly and characterization of biological components, real modularity is still a major research issue. The success of the bottom-up approach strictly depends on the clear definition of the limits in which biological functions can be predictable.

**Results:**

The modularity of transcription-based biological components has been investigated in several conditions. First, the activity of a set of promoters was quantified in *Escherichia coli* via different measurement systems (i.e., different plasmids, reporter genes, ribosome binding sites) relative to an *in vivo* reference promoter. Second, promoter activity variation was measured when two independent gene expression cassettes were assembled in the same system. Third, the interchangeability of input modules (a set of constitutive promoters and two regulated promoters) connected to a fixed output device (a logic inverter) expressing GFP was evaluated. The three input modules provide tunable transcriptional signals that drive the output device. If modularity persists, identical transcriptional signals trigger identical GFP outputs. To verify this, all the input devices were individually characterized and then the input-output characteristic of the logic inverter was derived in the different configurations.

**Conclusions:**

Promoters activities (referred to a standard promoter) can vary when they are measured via different reporter devices (up to 22%), when they are used within a two-expression-cassette system (up to 35%) and when they drive another device in a functionally interconnected circuit (up to 44%). This paper provides a significant contribution to the study of modularity limitations in building biological systems by providing useful data on context-dependent variability of biological components.

## Introduction

Standardization of components, abstraction, modularity and predictability are the main engineering principles for which the emerging field of Synthetic Biology lays the foundations [1], [2]. Following these principles, the ultimate goal is to design and construct new biological systems that exhibit a predictable and user-defined behaviour, starting from a set of quantitatively characterized standard components, exactly as it is accomplished in all the other fields of engineering [3], [4]. Standard biological parts, such as BioBricks, a trademark of The BioBricks Foundation [5], are the building blocks that may enable the composition of these systems, according to their abstraction hierarchy and standardized assembly process [6], [7]. The Registry of Standard Biological Parts [8] encloses a collection of thousands of on-line browsable BioBricks whose structures and functions are listed and, when properly characterized, they could be exploited for the bottom-up construction of the desired composite systems [9]. Such paradigm may contribute to the construction of biological solutions for a large variety of applications, from medicine to renewable energy production [10], [11].

However, the tremendous potential of Synthetic Biology is limited by the intrinsic complexity of living systems [12]. In fact, although abstraction and physical standardization concepts have been successfully proposed by the definition of BioBricks, exhaustive characterization of components is currently a major challenge [13]. Standard measurement techniques have been introduced for promoters, terminators and ribosome binding sites (RBSs) [14], [15]. In the case of promoters, the Relative Promoter Unit (RPU) method was proposed to improve the reproducibility of measurements among different instruments and different labs. This method relies on the measurement of the activity of promoters relative to a standard reference promoter, assayed *in vivo* in the same experimental conditions. RPUs have been recently used in the literature to characterize many promoters [16], [17] and they are also very popular among the international Genetically Engineered Machine (iGEM) competition community, as they were exploited by many teams to share their quantitative measurements in the Registry [18].

The central challenge about quantitative characterization is the modularity of components [19]. Modularity is a crucial aspect in the whole engineering world, as it enables to achieve predictable outcomes when different functional parts are interconnected [20]. Even if the successful interconnection of biological modules has been reported in many studies [13], [21], most of them relied on trial-and-error approaches or time-consuming debugging of the constructed systems [15], [22], [23] and nowadays the behaviour of composite parts is hard to predict from individually characterized components. Recent efforts in such research have focused on model-based design and qualitative prediction of genetic circuits behaviour [13], [24].

Crosstalk or incompatibility among components [1], context-dependent behaviour of parts [25], intrinsic noise that characterizes biological processes [26] and nonlinear effects on gene expression caused by cell machinery overburdening [27] are some of the limiting factors that contribute to the unpredictability of bottom-up-composed systems. For example, promoters activity may be affected by their flanking sequences and this contributes to the unpredictability of their behaviour when moved to a different physical context [25].

As Synthetic Biology key concepts are based on engineering, the success of this emerging field depends on the definition of the working boundaries in which biological functions can be predictable, in order to enable the bottom-up composition of customized systems. Recently, some experimental studies have been reported to assess modularity of biological components. Davis et al. [25] proposed design rules to engineer insulation of promoters in order to improve their predictability when different DNA sequences surround them. Hajimorad et al. [27] reported the working limits in which the superposition of the effects could be valid in a model system composed by one to three independent gene expression cassettes as a function of the system copy number. Both works are promising starting points for studying context-dependent activity of biological parts and devices.

Here, the modularity of transcription-based biological parts and devices has been investigated in *ad-hoc* constructed model systems. To study the context-dependent variability of biological parts, the quantitative behaviour of promoters was studied in three increasingly complex conditions in *E. coli*, a popular chassis in Synthetic Biology. First, the activity of a set of promoters was quantified *in vivo* via different biological measurement systems (i.e., different plasmids, reporter genes and ribosome binding sites) relative to a reference promoter in identical conditions, using the Relative Promoter Unit (RPU) approach. Second, promoter activity variation was measured when two independent gene expression cassettes were assembled in the same system. Third, the modularity of input devices was tested in a functionally interconnected framework, composed by a variable input device connected to a fixed output device (a logic inverter) expressing GFP.

## Results

### Characterization of a Set of Promoters Via Different Biological Measurement Systems

**Figure 1:**
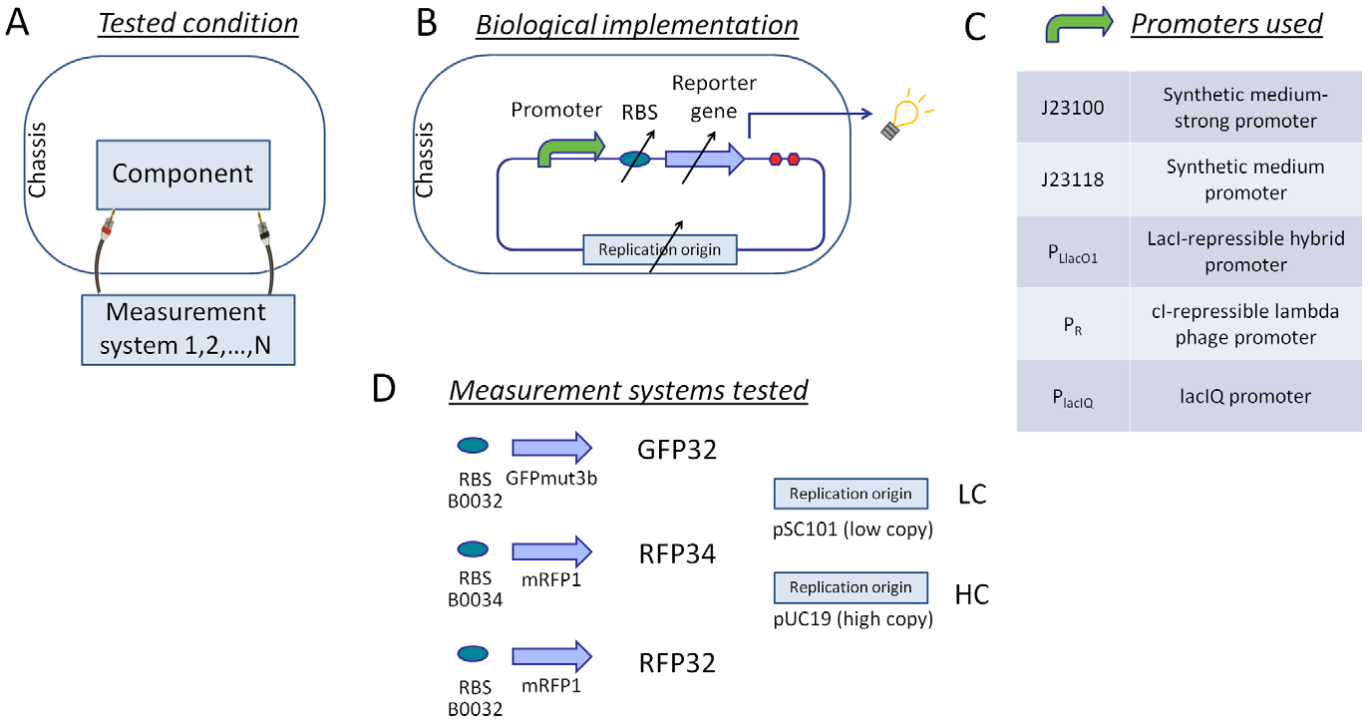
Study of individual promoters activity quantified via different measurement systems. A) Schematic functional representation of the tested framework. B) Promoters are tested via different biological measurement systems (i.e., RBSs, reporter genes and/or plasmid copy number) and their activity is computed relative to a standard reference promoter with the RPU approach. C) Promoters used in this study. D) Three reporter devices (GFP32, RFP34 and RFP32) and two copy number conditions (LC or HC) are used as different biological measurement systems.

In order to estimate the activity variation when a promoter is characterized via different biological measurement systems, a representative set of five widely used promoters was assembled to three different reporter expression devices in two different plasmid vectors and characterized in terms of RPU in the TOP10 strain (see Figure 1A and 1B). Relying on relative measurements, RPUs enable the comparison among different fluorescent proteins, used as reporters. A promoter quantified via different measurement systems should give the same results among the conditions. The considered promoters and tested conditions are listed in Figure 1C and 1D.

**Figure 2:**
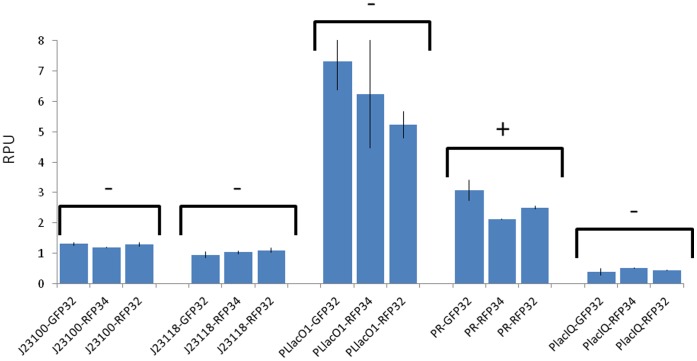
Measured RPU values for the five investigated promoters. Promoters were individually characterized via three reporter devices: GFP32, RFP34 and RFP32. Error bars represent the standard deviation of the mean activity computed on three clones. For each promoter, statistical analysis was performed via ANOVA test to compare the RPU activities measured via the three different reporter devices. Promoters showing a statistical difference (P<0.05) in the mean activities among the three conditions are marked with a ‘+’ sign, while promoters not showing any significant difference (P
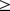
0.05) are marked with a ‘-’ sign. Strains with 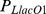 were induced with 1 mM of isopropyl 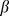-D-1-thiogalactopyranoside (IPTG).

Results are shown in Figure 2 for the low copy condition. Promoters span a >10-fold RPU range, in which 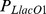 is the strongest one, while 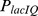 is the weakest one. Given a measurement system, the quantified activity of each promoter is reasonably reproducible among the technical replicates, giving an average coefficient of variation (CV) of 9%. As expected, promoters characterized via RFP34 give a higher absolute activity than with RFP32 (data not shown), as the BBa_B0032 RBS is weaker than BBa_B0034 [8], [15], [28]. Only 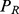 activity is statistically different among the three tested measurement systems (P<0.05, ANOVA), yielding a CV of 22% among the three measured mean activities.

The observed variability may be caused by downstream sequence-dependent promoter activity change. The maximum activity variability found in this set of promoters is relatively low and it is smaller than previously reported in other downstream sequence-dependent case studies [16], [25].

Promoters with GFP32 and RFP34 reporter devices were also tested in a high copy number plasmid. Results showed that the RPU activity of the strongest promoters (P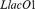 and P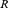) is much weaker in high copy than in low copy context (up to 4.4- and 2.3-fold respectively, see Figure S1 and Text S1). Such results are in accordance with other studies in which devices in high copy number showed saturation effects in strong promoters activity [17] or a nonlinear response in the DNA-mRNA device transfer curves [27].

### Promoter Activity Variation when Independent Expression Modules are Physically Combined

**Figure 3:**
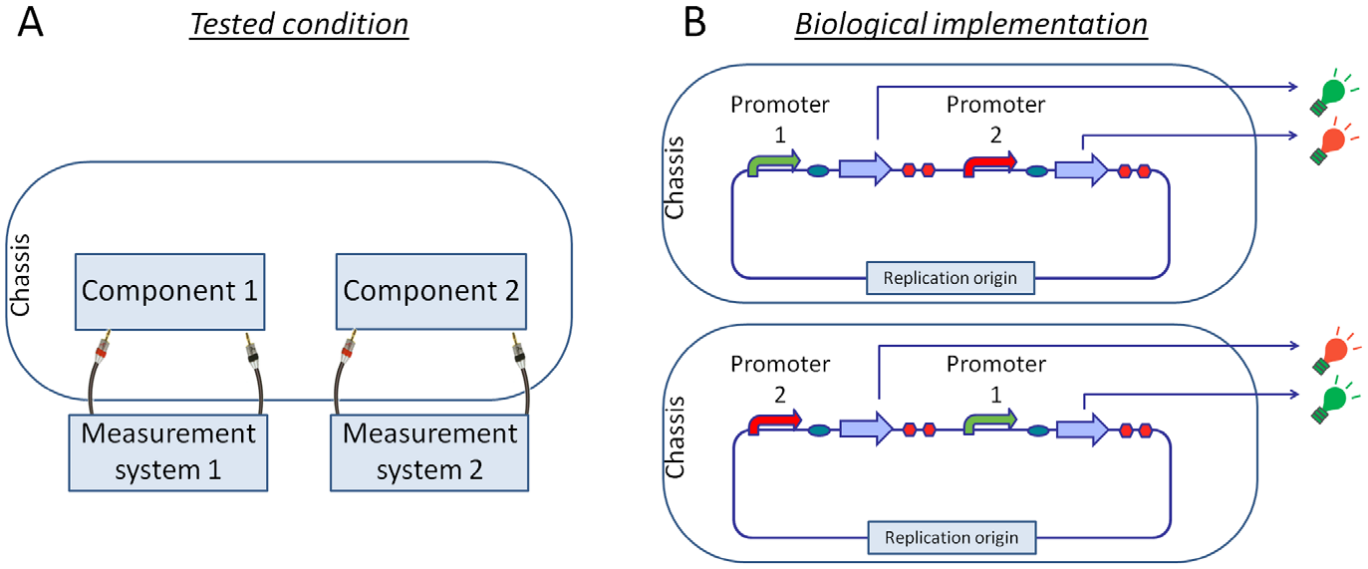
Study of promoters activity when two independent expression modules are physically combined. A) Schematic functional representation of the tested framework. B) Promoters with GFP32 or RFP34 are assembled in the same plasmid and quantified. The resulting activity will be compared to the one measured in the individual characterization study to investigate context-dependent activity changes. The two gene expression cassettes are also cross-exchanged to investigate the relative position-effect.

A subset of the reporter expression cassettes tested above were assembled in pairs in order to study the context-dependent variability in promoters activity when the system is slightly more complex. In this framework, constructs are combined in the same plasmid, but do not functionally interact with each other (see Figure 3A and 3B). In order to quantify the activity of two promoters in the same system, two reporter genes (GFP and RFP) had to be used. GFP32 reporter device driven by the medium-strength J23118 promoter was kept constant in all the constructed composite systems, while RFP34 reporter device was driven by one of the other four promoters (J23100, 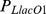, 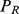 or 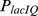). The relative position of the cassettes was also cross-exchanged (see Figure 3B).

**Figure 4:**
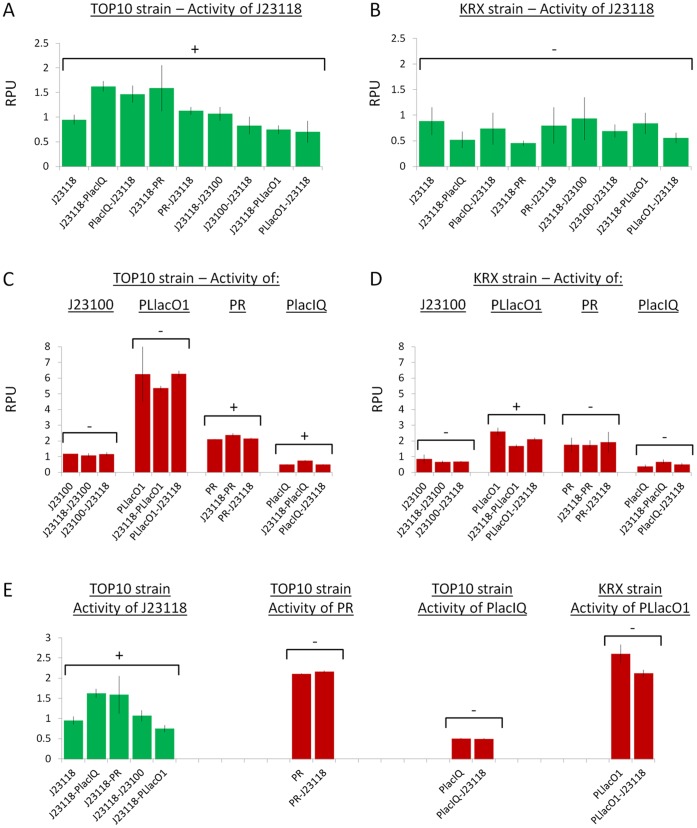
Measured RPU values for promoters assembled in the same plasmid in TOP10 and KRX strains. Panels A-D show the measured promoters activities in all the tested conditions. Green and red bars indicate that the promoter has been characterized via GFP32 and RFP34 reporter device respectively. Error bars represent the standard deviation of the mean activity computed on three clones. For each group, the RPU value of the individually characterized promoter is also reported. Statistical analysis was performed via ANOVA test to compare the RPU activities measured in the tested conditions. Promoters showing a statistical difference (P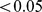) in the mean activities among the tested conditions are marked with a ‘+’ sign, while promoters not showing any significant difference (P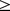 0.05) are marked with a ‘-’ sign. When a significant difference is present for a given promoter, the statistical analysis was also performed by excluding the conditions where the promoter drives the ‘downstream’ cassette and panel E shows the subset of conditions used for such comparisons, as well as the statistical analysis results. Strains with 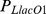 were induced with 1 mM of isopropyl 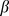-D-1-thiogalactopyranoside (IPTG).

Figure 4 shows the resulting activity of combined promoters in two different *E. coli* strains (see panels A-D). The activity of the individually characterized promoter, via the same measurement system, is also reported for each group. Among the groups where at least one of the mean activities showed statistical difference (P<0.05, ANOVA), CVs of 33%, 7%, 33% (for J23118, 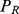, 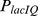 in TOP10) and 27% (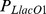 in KRX) were observed.

Part of the variability may be due to upstream sequence-dependent promoter activity change. In particular, the promoter driving the ‘upstream’ expression cassette has the same flanking sequences as the one in the individually characterized condition (i.e., the same vector sequence upstream and the same reporter expression device downstream), but the promoter driving the ‘downstream’ cassette has a different upstream sequence (i.e., the transcriptional terminator of the other cassette). If the data of the promoters driving the ‘downstream’ cassette are excluded from the variability analysis (see Figure 4E), only the J23118 promoter in TOP10 shows a significant difference among the tested contexts, with a CV of 35%. This demonstrates that flanking sequences significantly contribute to context-dependent differences, but they are not sufficient to explain all the observed variability.

### Modularity Test for Input Devices Connected to a NOT Gate

**Figure 5:**
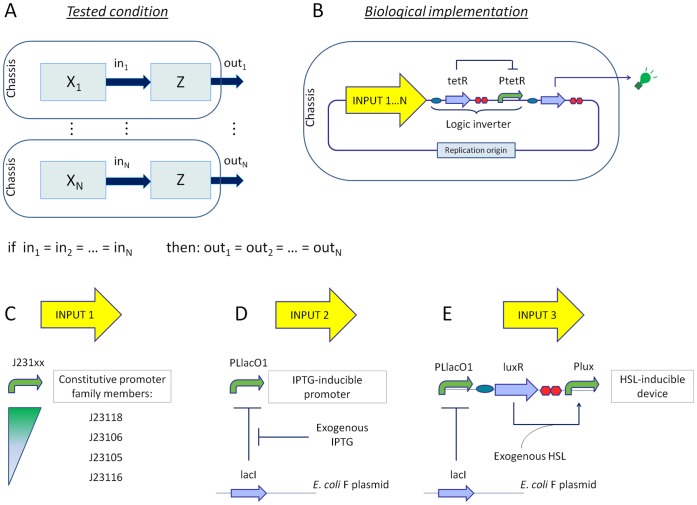
Modularity study for input modules in interconnected systems. A) Schematic functional representation of the tested framework. B) Different input devices are assembled upstream of a tetR-based logic inverter. They provide transcriptional signals that drive the inverter. C) INPUT1: a set of four constitutive promoters of different strengths. D) INPUT2: 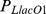 promoter, which is repressed by the endogenously-overexpressed lacI and can be induced by IPTG. E) INPUT3: luxR-based HSL-inducible device. 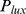 can be induced by LuxR-HSL complex. The luxR gene is produced by the weak basic activity of repressed 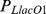 in absence of IPTG. IPTG = isopropyl 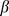-D-1-thiogalactopyranoside; HSL = N-3-oxohexanoyl-L-homoserine lactone.

The modularity of biological devices was studied when dealing with functionally interconnected circuits. The basic idea driving this part of the study is illustrated in Figure 5A. Considering interconnected systems composed by different input blocks (X_1_, X_2_, …, X*_N_*) and a fixed output block (Z) downstream, if the signals provided by the input blocks are the same (in_1_ = in_2_ = … = in*_N_*), the output signals should be identical (out_1_ = out_2_ = … = out*_N_*) even if the input blocks are structurally different.

To test this condition, the model systems shown in Figure 5B were constructed (with the inputs reported in Figure 5C–E) and tested in the KRX *E. coli* strain, which over-expresses the lacI repressor. The circuits are composed by different input devices interconnected to the same output device. The considered input modules were: i) a set of constitutive promoters of different strengths (four J231xx-family members, here called INPUT1), ii) a lacI-regulated promoter (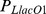, here called INPUT2) and iii) a luxR-regulated promoter (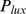, here called INPUT3). They provide a transcriptional signal that drives the output device. The latter is a logic inverter (or NOT gate), i.e. a promoterless tetR repressor-expressing cassette connected with a tetR-repressible promoter (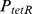) downstream that can be inhibited by TetR. 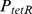 expresses GFP as the system output through the GFP32 reporter device. The lacI-, luxR- and tetR- regulated systems, as well as the J231xx constitutive promoters, are all widely used in the design of genetic circuits [16], [29], [30]. If modularity persists, identical transcriptional signals should trigger identical GFP outputs for any connected input device.

**Figure 6:**
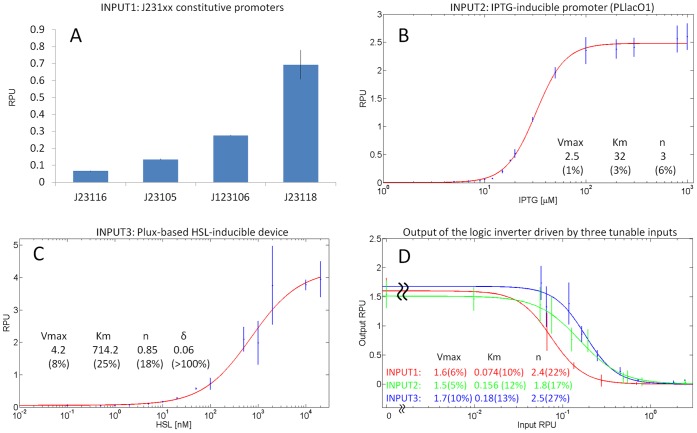
Individual characterization of the three investigated input modules (A, B, C) and logic inverter transfer function when driven by the different inputs (D) in KRX strain. Input modules were individually characterized in terms of RPU via RFP34, while the logic inverter was measured via GFP32. Data points are the mean activities computed on three clones and error bars represent the standard deviations. Induction and input-output curves were fitted (solid line) and identified parameters are reported with their estimated CV in brackets. The 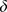 parameter of INPUT2 and output curves was fixed to zero.

To assess this hypothesis, at first the input devices were individually characterized in terms of RPU via RFP measurements. Figure 6A–C report the characterization of the individual input devices. The four considered constitutive promoters yielded a 10-fold activity range (Figure 6A). The induction curves of the two inducible inputs showed that they were tightly controlled (i.e., the basic activity of promoters in the uninduced state was very low) and they could be regulated over a wide range of activities, yielding a maximum activity of 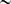
2.5 RPUs (lac) and 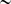
4.2 RPUs (lux). Their induction curves were fitted with a Hill function (Figure 6B and 6C).

Subsequently, the interconnected systems composed by the different input devices and the NOT gate downstream were considered. The transcriptional activity of the different inputs was tuned either by changing the constitutive promoter (INPUT1) or by exogenously adding different inducer amounts to the bacterial cultures (IPTG for INPUT2 and HSL for INPUT3). The response of the logic inverter output device was measured in terms of RPU via GFP, thus yielding an input-output transfer function for each of the three input devices used. If the modularity hypothesis is valid, the three transfer functions should be identical, as input systems should be functionally interchangeable. Figure 6D shows the three resulting input-output curves for the logic inverter, as well as the identified parameters of the curve fitting. The V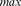 and *n* values showed a modest variation among the three conditions (CV of 6% and 17% respectively), while the K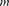 values showed a higher variation (CV of 44%). Such difference of the switch point-related parameter may be caused by the input promoters activity variation from the individually (characterized) context to the interconnected circuit.

## Discussion

The apparent unpredictability of genetic circuitry is one of the five *hard truths*, recently captured as the major challenges for Synthetic Biology, that nowadays prevent the fully rational design of biological systems following engineering principles [12]. When parts are put together, they may not behave as expected for several reasons, like the incompatibility of two or more parts or the excessive overloading of transcriptional/translational machinery of the host cell. The term ‘retroactivity’ has also been introduced to define the unwanted characteristics change of a component upon interconnections. This phenomenon is currently under investigation by a number of research groups [20], [31]. Software tools have been developed that aid the bottom-up design of genetic circuits and improve their predictability [32]. Experimental studies of context-dependent variability and modularity have also been reported for biological parts to elucidate the predictability boundaries of components behaviour [25], [27]. The aim of this work was to expand such experimental investigations by providing useful data on context-dependent variability of transcription-based components and testing the modularity of devices with simple *ad-hoc* constructed model systems in *E. coli*. The RPU approach was applied in order to generate reproducible results among the experiments and to enable the sharing of the data presented in this work, as they are expressed in standard measurement units. To compute RPU, absolute promoters activity was divided by the absolute activity of a reference promoter.

The first preliminary goal was to estimate the activity variation for a set of five popular promoters when measured in TOP10 strain via different measurement systems: three different reporter devices were assembled to the promoters in a low copy vector and were used to quantify their activity *in vivo*. The reference promoter was quantified via the same measurement system, so if promoters activity does not change with the reporter device assembled downstream, the studied promoters should theoretically yield the same RPU activity values when measured via different devices. Only one of the five promoters showed a significant activity difference among the three tested reporter devices, yielding a CV of 22%. Plasmid copy number dependence was also studied for the tested promoters and results showed that the RPU activity of the strongest promoters is significantly weaker (up to 4.4-fold) in high copy than in low copy context. This result is in accordance with other studies [17], [27]. It confirms the unsuitability of high copy vectors for the characterization of biological parts, because the activity of components with a high energy demand could be underestimated when present in a huge number of copies per cell.

The second goal was to estimate how much the RPU activity changes when a promoter with a given reporter device is moved from the individual context to a complex system composed by two non-interacting gene expression cassettes in the same plasmid. Results showed a maximum activity variation of 33% and 27% for TOP10 and KRX strains respectively.

In these two studies, some of the activity variations could be explained by the physical un-insulation of promoters, in fact it is known that upstream and downstream sequences can significantly change the activity of a promoter. This can explain the variation in the RPU activity of individually characterized promoters when the downstream reporter device is varied; it can also explain the activity variation of a promoter (relative to the individual characterization case) when the upstream sequence is changed by assembling another expression cassette in this position. However, not all the observed variability could be explained by un-insulation, in fact, considering the systems with two combined cassettes in the same plasmid, the activity of the promoter driving the ‘upstream’ cassette could differ from the one in the individually characterized context, even if the promoter had the same upstream and downstream sequences. Such effect has also been found by Hajimorad et al. [27] while testing the superposition of the effects of different independent gene expression cassettes in the same plasmid.

The third goal of this work was to evaluate the interchangeability of different input modules driving the same output device. The transcriptional signals generated by the investigated input components were measured in terms of RPU via RFP and then the components were used to regulate a tetR-based logic inverter with GFP downstream. Three input modules were tested: four constitutive promoters of different strengths (considered as one module, transcriptionally tunable by changing the promoter itself), an IPTG-inducible promoter and an HSL-inducible device. In a modular framework, the three steady-state input-output transfer functions of the logic inverter should be the same, as devices driven by identical signals should yield identical outputs. However, although the obtained input-output curves had similar shapes (and substantially confirmed the modularity hypothesis), their switch point showed a CV of 44% among the conditions. One or more of the above mentioned factors could be responsible for this variability, e.g. the different RBS-gene downstream of the promoter in characterization and test stages could cause a downstream sequence-dependent activity change of the input.

Previously published works also reported successful modularity of input devices in interconnected systems, such as logic gates, actuators or feed-forward circuits [13], [24], [28], [33]. Although they proved the correct functioning of complex and valuable systems, the modularity assessment often relied on qualitative behaviour comparisons, without providing variability indexes for quantitative comparisons. The overall variability results of this work suggest that increasingly complex conditions yield an increasing variability in components activity. Designers of synthetic biological systems must take into account such variability entity when dealing with the bottom-up composition of systems. In particular, because promoters activity may vary when characterized via different measurement systems and when parts are placed in composite circuits, this uncertainty and its expected entity should be considered in the design process. Simulations of deterministic or stochastic mathematical models and sensitivity analysis on their parameters can help handling the variability in the described contexts, with the final goal of improving the robustness of the designed solution. According to design specifications, different biological systems may tolerate different variability levels of their components activity, so the significance of the variability entities encountered in this work and in other case studies depends on the specific target system. The conditions investigated in this study are recurrent in the construction of synthetic biological systems: a promoter from a catalogue of well-characterized parts can be chosen to regulate an individual gene of interest; non-interacting gene expression cassettes can be combined to form high-order composite systems; interconnection of devices is highly important in the tuning of gene expression, as the optimization of gene dosage is a major task that should be predictably achieved by means of different constitutive and inducible promoters.

The use of M9 supplemented medium in the performed characterization experiments most probably contributed to the reproducibility of many results obtained in this study, as it is a standard growth medium with an extremely low background fluorescence. Medium composition can significantly affect gene expression and protein synthesis rates, so experiments performed in different growth media can yield highly different outcomes [14]. However, because promoter activity in all the recombinant strains has always been measured relative to a standard reference promoter assayed in the same conditions, the use of different growth media should give similar results and the possible differences among the media may be due to nonlinear phenomena in transcription/translation machinery or to unwanted interactions between promoter and specific molecules present in the medium [14], [34]. To support these comments, Figure S3 shows the RPU activities of the five promoters characterized via different measurement systems in LB medium and the results are in full accordance with the ones obtained in M9 supplemented medium (see Figure 2). In fact, results demonstrated that, as it was observed with M9 supplemented medium, only for the 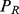 promoter the mean activities showed a statistical difference among the measurement conditions, with a CV of 17%. Moreover, the RPU values obtained in LB medium are consistent with the ones obtained with the M9 supplemented medium.

Finally, it is worth noting that in the model systems considered here, promoters have very similar predicted transcription start sites, so, given a specific downstream device, the produced mRNA should be the same for all the promoters [14], [28], [35]. All the tested cultures had a growth rate similar to the strain bearing the standard reference promoter (data not shown). Moreover, the mRFP1, GFPmut3b and tetR genes, used to characterize promoters and to interface devices, have comparable lengths. Taken together, these features contribute to the simplification of the designed model systems, while deviations from these conditions still have to be tested and may yield higher context-dependent variability. In general, nonlinearities in components activity can be function of these and other factors, like the codon usage of the expressed genes, promoters/RBSs strength and bacterial strain used, so similar studies should be conducted to investigate such factors.

## Materials and Methods

### Plasmid Construction and Cloning

BioBrick™ Standard Assembly was used to construct all the plasmids of this study, following a number of conventional molecular biology techniques. As a result, all the DNA junctions between parts had the TACTAGAG sequence (or TACTAG when the downstream part was a coding sequence) [36]. DNA-modifying enzymes were purchased from Roche. DNA purification kits were purchased from Macherey-Nagel. Chemically competent TOP10 (Invitrogen) were cultivated in LB medium and were used as hosts for plasmid propagation, except for pSB4C5(BBa_I52002) which was propagated in chemically competent DB3.1 (Invitrogen), a ccdB toxin-tolerant strain. Ampicillin (100 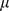
g/ml), Chloramphenicol (12.5 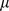
g/ml) or Kanamycin (50 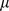
g/ml) were added as required.

TOP10 and KRX (Promega) strains were used as chassis for all quantitative experiments. Enzymes, purification kits and competent cells were used according to manufacturer's instructions. All the plasmids realized in this study were assembled from basic or composite parts from the Registry 2009 or 2010 DNA Distribution [8]. Table 1 reports the basic BioBrick™ parts used to design the characterized systems, while Figure S2 shows the detailed composition of all the systems. If not differently stated, all the constructs were tested in the pSB4C5 low copy vector (pSC101 replication origin), maintained in transformants by adding 12.5 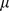
g/ml of Chloramphenicol to the growth media. Long-term stocks, routinely stored at −80°C, were prepared for all the recombinant strains by mixing 250 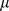
l of 80% glycerol with 750 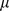
l of bacterial cells grown in selective LB.

**Table 1:** List of parts used to design the model systems.

BioBrick™	Description
BBa_B0015	double transcriptional terminator
BBa_B0031	weak RBS
BBa_B0032	medium-weak RBS
BBa_B0034	strong RBS
BBa_C0040	TetR repressor coding sequence
BBa_C0062	LuxR activator coding sequence
BBa_E0040	GFPmut3b coding sequence
BBa_E1010	mRFP1 coding sequence
BBa_I14032	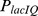 constitutive promoter
BBa_J23100	Constitutive promoter family member
BBa_J23101	Standard reference promoter
BBa_J23105	Constitutive promoter family member
BBa_J23106	Constitutive promoter family member
BBa_J23116	Constitutive promoter family member
BBa_J23118	Constitutive promoter family member
BBa_R0011	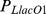 synthetic promoter
BBa_R0040	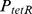 synthetic promoter
BBa_R0051	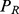 promoter from lambda phage
BBa_R0062	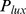 promoter from *Vibrio fischeri*

### Promoter Characterization

Recombinant bacteria from a long-term glycerol stock were streaked on an LB agar plate and grown for about 20 hours at 37°C. 1 ml of selective M9 supplemented medium (M9 salts, 1 mM thiamine hydrochloride, 0.2% casamino acids, 2 mM MgSO4, 0.1 mM CaCl2, 0.4% glycerol) was inoculated with a single colony from the streaked plate and incubated at 37°C, 220 rpm shaking for about 20 hours. Bacteria were diluted 1∶500 in 2 ml of fresh selective M9 supplemented medium and grown for 6 hours under the same conditions as before. When required, properly diluted isopropyl 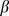-D-1-thiogalactopyranoside (IPTG, Sigma Aldrich, #I1284) or N-3-oxohexanoyl-L-homoserine lactone (HSL, Sigma Aldrich, #K3007) was added to the liquid culture after 3 hours from the 1∶500-dilution (if not differently stated) to reach the desired final concentration of inducers. For each culture, a 200-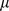
l aliquot was transferred into a flat-bottom 96-well microplate (Greiner) and assayed for about 6 hours in an Infinite F200 microplate reader (Tecan) with a kinetic cycle programmed with the i-control software (Tecan). Fluorescence (Ex:485 nm, Em:540 nm for GFP; Ex:535 nm, Em:620 nm for RFP) and absorbance (600 nm) were measured every 5 minutes. In every measurement cycle, cultures were shaken (linear shaking, 3 mm amplitude) for 15 seconds and then, after a 5-second wait time, the measurements started. Temperature was kept constant at 37°C during all the experiment. The gain of GFP and RFP fluorescence measurements was set at 50 when assaying reporter genes on high copy vectors, while it was set at 80 for low copy assays, in which the fluorescence signal is weaker. The absorbance of sterile M9 medium and the autofluorescence of the strain without fluorescent proteins were measured in order to estimate the absorbance and fluorescence background, respectively. In each experiment, bacteria bearing the standard reference promoter BBa_J23101 driving a reporter gene (here called *reference culture*) were also assayed, so that strain, plasmid copy number, antibiotic resistance, reporter gene and RBS were exactly the same as in the cultures of interest.

### Data Analysis

Data were analyzed as described in [14] to obtain RPUs. Briefly, M9 and strain background values were subtracted to all the absorbance and fluorescence raw measurements respectively to obtain values proportional to the per-well cell count and number of fluorescent proteins. The reporter protein synthesis rate per cell time series (S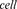) of each culture was computed as the numeric time derivative of the fluorescence values, divided by absorbance. This time series was averaged in the exponential growth phase for each well, thus yielding 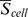. The RPU value of a promoter was computed as 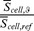, where *ϕ* is the culture bearing the promoter of interest and *ref* is the reference culture.

The induction curves of the regulated input devices and the input-output transfer curves of the logic inverter were fitted with the Hill function 
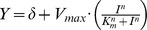
 or 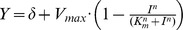, where I can be an inducer molecule concentration or an RPU input, 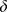
+V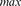 is the maximum RPU output, K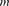 is the input that yields Y = 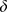
+V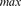/2, 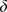 is the basic RPU activity of the promoter in the OFF state and *n* is the Hill coefficient. All the data were processed either with Microsoft Excel 2007 or with the MATLAB 2007b suite (MathWorks, Natick, MA). In particular, the fitting of the Hill functions was performed through the MATLAB *lsqnonlin* routine which implements the least squares method.

All the coefficients of variation were corrected for small samples: for N samples, 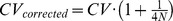. Hypothesis tests were performed via MATLAB.

To assess the statistical difference among the mean promoter activity values in a group, ANOVA test was performed. If a difference was detected in the group, individual t-tests were performed to compare the mean values of the group members to identify statistically different sub-groups in order to compute the CV. The p-values (P) were corrected for multiple comparisons with the Bonferroni method. The mean values of the non-significantly different sub-groups were averaged and the final CV within the group was computed on the mean values of the statistically different sub-groups. If ANOVA highlighted a statistical difference, but multiple comparisons showed no evident sub-groups, the CV was computed on all the mean activities among the group.

## Supporting Information

Figure S1
**Measured RPU values for individual promoters characterized in a high or low copy vector.** Promoters were characterized via GFP32 (panel A) and RFP34 (panel B) reporter devices. Error bars represent the standard deviation of the mean activity computed on three clones. Strains with 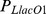 were induced with 1 mM of IPTG.(EPS)

Figure S2 **Composition of the constructed plasmids.** BioBrick™ composition is shown for the plasmid inserts used in this paper to study the context-dependent variability of promoters as a function of the biological measurement system (A), when independent expression cassettes are assembled in the same plasmid (B) and in an interconnected genetic circuit containing a logic inverter (C). All the shown inserts were cloned and tested in the pSB4C5 vector backbone and all the inserts of panel A were also cloned and tested in pSB1A2. Green, red and yellow groups in panel A include the promoters that have been individually tested with the GFP32, RFP34 and RFP32 reporter device respectively. Cyan group in panel B includes the constructs in which two independent expression cassettes are tested, while the grey group in the same panel includes the individual expression cassettes. Violet group in panel C includes the measurement constructs of the input devices (INPUT1, INPUT2 and INPUT3), while the pink group in the same panel includes the model systems where input devices are interconnected with a logic inverter. Inserts highlighted with dashed line in the three panels represent the reference constructs, containing the BBa_J23101 promoter with an appropriate reporter device downstream, used to compute the RPUs of the promoters of interest.
(EPS)

Figure S3
**Measured RPU values for the five investigated promoters in LB medium.** Promoters were individually characterized via three reporter devices: GFP32, RFP34 and RFP32 in pSB4C5 vector backbone in TOP10 strain grown in selective LB medium. Error bars represent the standard deviation of the mean activity computed on three clones. For each promoter, statistical analysis was performed via ANOVA test to compare the RPU activities measured via the three different reporter devices. Promoters showing a statistical difference (P<0.05) in the mean activities among the three conditions are marked with a ‘+’ sign, while promoters not showing any significant difference (P
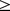
0.05) are marked with a ‘-’ sign. Promoters were assayed through the same protocol described for M9 supplemented medium, with the following exceptions: i) cultures were grown for 3 h instead of 6 hours before being transferred into the microplate; ii) strains with 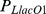 were induced with 1 mM of IPTG 1.5 hours before being transferred and iii) the gain of GFP fluorescence was always set at 50 instead of 80.(EPS)

Text S1 **Crosstalk estimation between GFP and RFP spectra; preliminary design of the interconnected system; characterization of individual promoters in a high copy vector.**
(PDF)
