# Correction: Bottom-Up Engineering of Biological Systems through Standard Bricks: A Modularity Study on Basic Parts and Devices

**DOI:** 10.1371/annotation/91e7d3a1-2f50-4f84-8b12-2c21f88438c3

**Published:** 2012-10-30

**Authors:** Lorenzo Pasotti, Nicolò Politi, Susanna Zucca, Maria Gabriella Cusella De Angelis, Paolo Magni

There is an error in Text S1. The correct file can be found here: 
