# Supplementary material for: Correction: Bottom-Up Engineering of Biological Systems through Standard Bricks: A Modularity Study on Basic Parts and Devices
Source: PLoS One. 2012 Oct 30;7(10):10.1371/annotation/91e7d3a1-2f50-4f84-8b12-2c21f88438c3. doi: 10.1371/annotation/91e7d3a1-2f50-4f84-8b12-2c21f88438c3 (PMC3502579; doi:10.1371/annotation/91e7d3a1-2f50-4f84-8b12-2c21f88438c3)
Supplement: Supplementary file 1 [file pone.91e7d3a1-2f50-4f84-8b12-2c21f88438c3.s001.pdf]

Text S1

Crosstalk estimation between GFP and RFP spectra

Crosstalk between fluorescent proteins spectra might be an issue regarding systems containing both GFP- and RFP-expressing devices. In order to validate whether this phenomenon was significantly present with the acquisition system used in this study (i.e., the Infinite F200 microplate reader, Tecan), at least in the tested conditions for the combined gene expression cassettes, an *ad-hoc* experiment was performed. TOP10 recombinant cultures bearing J23118 with GFP32, (the only GFP-expressing construct in the combined cassettes study), P_{LlacO1} with RFP34 and P_{lacIQ} with RFP34 (the strongest and the weakest RFP-expressing constructs), all in the low copy vector pSB4C5, were grown as described in the Methods section in the main text. Cultures bearing P_{LlacO1} with RFP34 were induced with 1 mM of IPTG. All the cultures were assayed in the microplate reader (gain=80) as described in the Methods section in the main text. In this experiment, red fluorescence was measured for J23118 with GFP32 and P_{lacIQ} with RFP34, while green fluorescence was measured for J23118 with GFP and P_{LlacO1} with RFP34.

After a proper background subtraction (see main text, Data analysis section), the $100 \cdot \frac{\overline{S}_{cell,J23118,GFP32}}{\overline{S}_{cell,PlacIQ,RFP34}}$ (red fluorescence) and the $100 \cdot \frac{\overline{S}_{cell,PLlacO1,RFP34}}{\overline{S}_{cell,J23118,GFP32}}$ (green fluorescence) ratios, estimating the maximum percent contribution that GFP and RFP could give to red and green fluorescence measurements respectively, were computed. No detectable RFP contribution could be observed in the green fluorescence acquisitions, while the resulting ratio was 1.4% when red fluorescence of GFP-expressing cells was acquired. This can be considered as reasonably low crosstalk value.

Preliminary design of the interconnected system

The first design of the interconnected system included a logic inverter with a strong RBS (BioBrick BBa_B0034) upstream of the tetR gene. Such system, however, always remained in the OFF state even when the uninduced P_{lux} promoter, whose basic activity was very low, was assembled upstream. Only the promoterless logic inverter gave a high output when tested (data not shown). This result is consistent with previous findings in which such logic inverter was tested in similar conditions [15]. In order to construct a logic inverter that could switch from the ON state to the OFF state in a range of RPUs between 0.05 and 2, which is exhibited by a number of easy-to-retrieve promoters from the Registry of Standard Biological Parts [14], the RBS upstream of tetR was changed. A much weaker candidate was chosen (BioBrick BBa_B0031). It gave the expected effect (see main text), as the switch point occurred when the input RPUs were $\sim$0.14.

The low copy vector condition was chosen to characterize the system because such condition can give more reliable results than in high copy vectors [17, 27]. Moreover, attempts in cloning the interconnected circuit in a high copy vector (pSB1A2, which has a pUC19-derived replication origin) gave no successful transformants, suggesting that one (or more) of the modules causes a high metabolic burden when present in >100 DNA copies.

A set of four synthetic constitutive promoters of different strengths was chosen as the INPUT1. All of them are 35-bp long and share a common structure [S1]. Inducible lacI- and luxR-regulated promoters were chosen as INPUT2 and INPUT3, respectively. Single-cell analysis reported in [17, 28, S2] showed that these two systems produce a homogeneous response in an induced cell population in presence of sub-saturating concentrations of IPTG or HSL. KRX *E. coli* strain was used in this study because it overexpresses the LacI repressor through a lacI expression cassette with the lacIq mutation, carried in the F plasmid. This allows the tight transcriptional control of lacI-regulated promoters without including a lacI gene in the circuit.

Characterization of individual promoters in a high copy vector

It is common knowledge that the activity of genetic parts in high copy number vectors can be nonlinearly affected by the overloading of cell machinery due to the high copy number of the DNA-encoded functions [17]. This was confirmed by comparing the activity of promoters characterized via the same reporter device in low copy and high copy vectors (see Figure S1): the RPUs of the two strongest promoters, P_{LlacO1} and P_R, were respectively 4.4- and 2.3-fold lower in high copy when compared to low copy. This means that, given a reporter device, the ratio between the activity of the promoter of interest and the reference is lower in high copy when compared to low copy. The other promoters did not show such a large difference (<1.3-fold). The observed large-entity variations could be due to saturation effects in transcription/translation processes that occur for the strongest promoters in high copy condition, while such effects were absent for the other (weaker) promoters.

Supporting references

S1. Anderson JC. J231xx promoter collection. Available: http://partsregistry.org/Part:BBa J23100. Accessed 2012 Jun 18.

S2. Khlebnikov A, Risa O, Skaug T, Carrier TA, Keasling JD. Regulatable arabinose-inducible gene expression system with consistent control in all cells of a culture. *Journal of Bacteriology*, 182(24):7029-7034, 2000.
